# Functional magnetic resonance imaging data of incremental increases in visuo-spatial difficulty in an adult lifespan sample

**DOI:** 10.1016/j.dib.2017.01.004

**Published:** 2017-01-11

**Authors:** Kristen M. Kennedy, Jenny R. Rieck, Maria A. Boylan, Karen M. Rodrigue

**Affiliations:** Center for Vital Longevity, School of Behavioral and Brain Sciences, The University of Texas at Dallas, Dallas, TX 75235, USA

**Keywords:** fMRI, Spatial judgment, Difficulty, Lifespan, Aging, BOLD

## Abstract

These data provide coordinates generated from a large healthy adult lifespan sample undergoing functional Magnetic Resonance Imaging (fMRI) while completing a spatial judgment task with varying levels of difficulty, as well as a control categorical condition. The data presented here include the average blood-oxygen-dependent (BOLD) response to the spatial judgment vs. the control task, as well as the BOLD response to incremental increasing difficulty; see also “Age-related Reduction of BOLD Modulation to Cognitive Difficulty Predicts Poorer Task Accuracy and Poorer Fluid Reasoning Ability” (Rieck et al., 2017) [1].

**Specifications Table**

**Table:** 

Subject area	Cognitive Neuroscience
More specific subject area	Functional Magnetic Resonance Imaging of spatial judgment
Type of data	Coordinate tables, figures
How data was acquired	Philips Achieva 3 T whole body scanner
Data format	Analyzed using Statistical Parametric Mapping 8
Experimental factors	
Experimental features	Participants performed a spatial judgment task in which they conducted two types of judgments. A categorical (LEFT/RIGHT) judgment was used as a control condition and a coordinate (NEAR/FAR) judgment was used with three levels of difficulty.
Data source location	Dallas, Texas, United States of America
Data accessibility	Data provided in article

**Value of the data**

• This dataset provides a sizable sample of healthy adults who performed a spatial judgment task.
• These data show differential BOLD responses for varying levels of visuo-spatial difficulty across the sample.
• The data provide specific MNI coordinates of brain regions evoked by the task.
• These data are potentially useful to investigators studying differences in fMRI activation to non-verbal, spatial stimuli across the adult lifespan.

## 1 Data

**Fig. 1:**
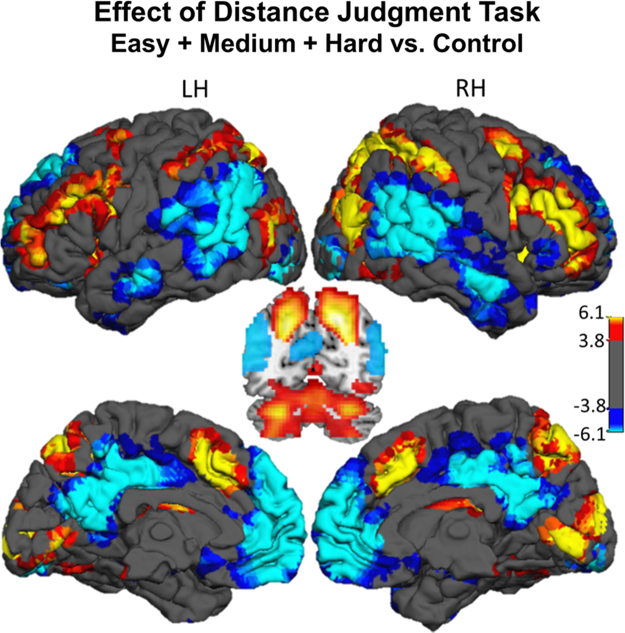
*Effect of Easy, Medium, and Hard Tasks vs. Control.* Hot blobs indicate regions in which there was greater activity during all levels (Easy, Medium, Hard) of the coordinate distance judgment task versus the coordinate control task. Cool blobs indicate regions in which there was greater activity during the left-right coordinate control condition. Color scale indicates *t*-values; *Abbreviations*: LH – Left Hemisphere; RH – Right Hemisphere.

**Fig. 2:**
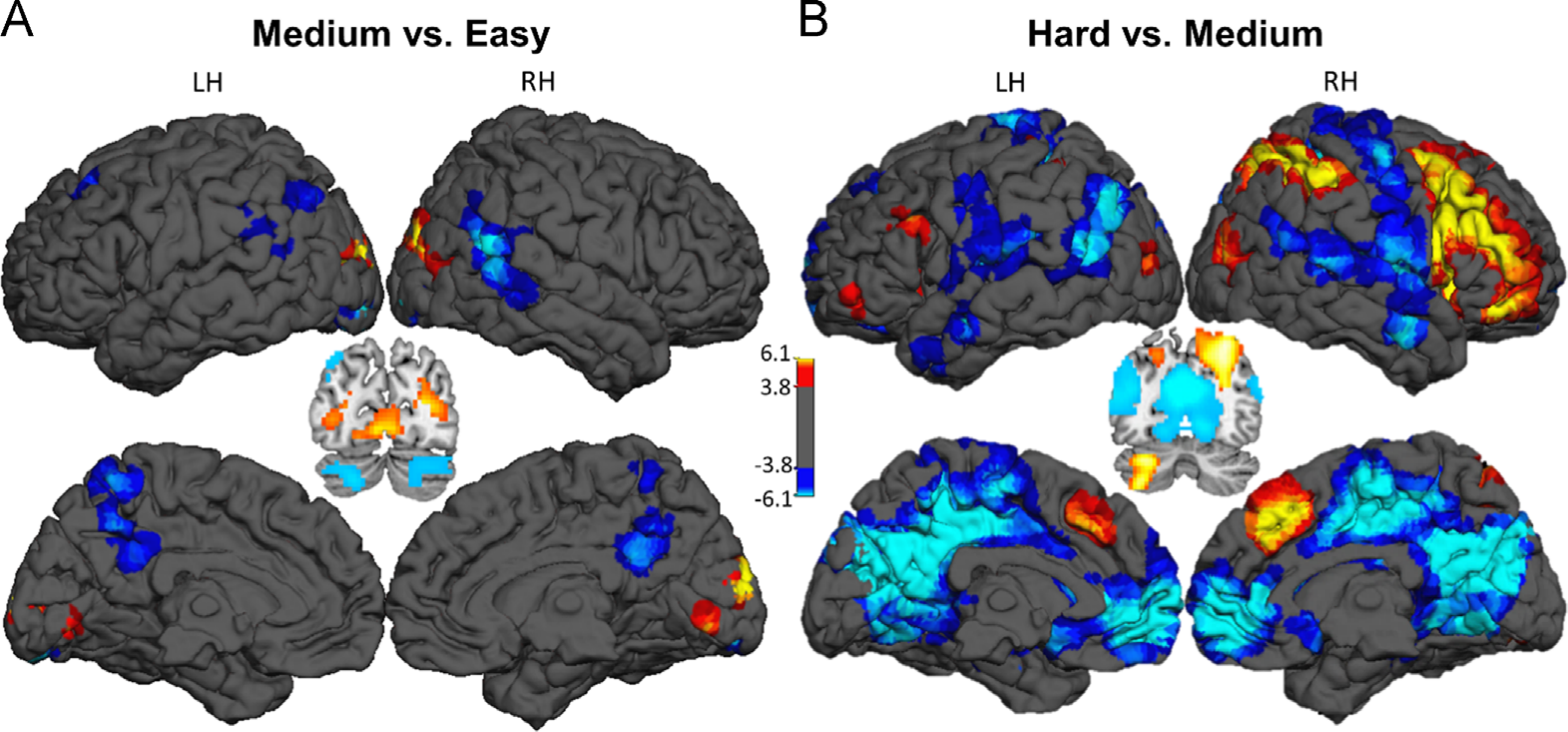
*Effect of incremental increasing difficulty across the entire sample*. Panel A shows the contrast of activation for Medium>Easy trials. Panel B shows the contrast of activation to Hard>Medium trials. Color scale indicates *t*-values. *Abbreviations*: LH – Left Hemisphere; RH – Right Hemisphere.

While undergoing fMRI, healthy adult participants completed a blocked-design spatial judgment task with three levels of difficulty (Easy, Medium, and Hard). These data have previously been analyzed with regard to age [1]. The data shown here represent the group level analyses examining the effect of the distance judgment task (Easy, Medium, Hard vs. Control – Table 1 and Fig. 1) as well as the effect of incremental increasing difficulty (Medium vs. Easy – Table 2 and Hard vs. Medium – Table 3, both shown in Fig. 2).

## 2 Experimental design, materials and methods

### 2.1 Participants

Participants included 161 healthy adults, ages 20–94 (mean age=51.93±18.9 years; 95 women; 66 men) who volunteered from the Dallas-Fort Worth area. Inclusion criteria for the study required that all participants be right-handed, fluent English speakers, and have normal or corrected-to-normal vision (at least 20/40). Participants were also screened for dementia using the Mini Mental State Examination (MMSE; [2]), with a cutoff of 26; volunteers were also required to have no history of neurological or psychiatric conditions, head trauma, drug or alcohol problems, or significant cardiovascular disease (however, *n*=32 with a self-reported diagnosis of hypertension). Participants were compensated for their time and informed consent was obtained in accordance with protocol approved by the University of Texas at Dallas and the University of Texas Southwestern Medical Center.

### 2.2 Experimental design

The data shared here are from a large lifespan dataset in which 161 healthy adults completed a blocked-design distance judgment task while undergoing fMRI. The spatial judgment task involved two types of judgments (modeled after [3] and [4]). The first type of judgment, which served as the control condition, required participants to make a categorical (LEFT/RIGHT) judgment. Participants saw a dot on the left or right side of a horizontal bar and had to indicate using a button press on which side of the bar the dot was present.

Participants also made a coordinate (NEAR/FAR) distance judgment which had three levels of difficulty: Easy, Medium, and Hard. First, participants saw a vertical reference line, next they were shown a horizontal line with a dot either above or below the line; the judgment required participants to determine whether the dot was “nearer to” or “farther from” the horizontal bar, given the previously seen vertical line. As difficulty increased, the distance between the dot and the horizontal line became harder to determine the “nearness” or “farness” compared to the reference line. A schematic of the task can be found in Fig. 1 of Rieck and colleagues [1]. Prior to the scanning session, participants completed a practice session to ensure that the participants were comfortable with the instructions. Each participant completed three runs of the task, resulting in ~15 min of scan time. The task was presented using PsychoPy v1.77.02 [5,6].

### 2.3 Image acquisition

Data were acquired on a single Philips Achieva 3 T whole body scanner using a 32-channel head coil. BOLD fMRI data were collected using a T2*-weighted echo planar imaging sequence in 29 interleaved axial slices parallel to AC-PC line, 64×64×29 matrix, 3.4×3.4×5 mm^3^, Field of View (FOV)=220 mm, Echo Time (TE)=30 ms, Repetition Time (TR)=1500 ms. High-resolution anatomical images were also acquired with a T1-weighted MP-RAGE sequence with the following parameters: 160 sagittal slices, 1×1×1 mm^3^ voxels; 256×204×160 matrix, FOV=256 mm, TE=3.8 ms, TR=8.3 ms, Flip angle=12°.

### 2.4 Image processing

Data from each individual were preprocessed using SPM8 (Wellcome Department of Cognitive Neurology, London, UK). Preprocessing included the following steps: slice time acquisition correction, motion correction, normalization, and smoothing (using an isotropic 8 mm^3^ full-width-half-maximum Gaussian kernel). In order to identify runs with motion outliers, ArtRepair [7] was used to determine potential outlier volumes for each participant. We examined all three runs for each participant, and runs that had more than 15% outlier volumes (~30 volumes) with greater than 3% deviation from the mean in global intensity spikes or greater than 2 mm of motion displacement were flagged. Five participants had one run with more than 15% percent outlier volumes, so that run was excluded.

At the individual subject level, BOLD response to each condition (Control, Easy, Medium, Hard) was modeled in SPM as a block convolved with a canonical hemodynamic response function; six directions of motion-estimates for each volume generated from ArtRepair were also included as nuisance covariates. Several contrasts of interest were computed at the individual level for subsequent analysis at the group level: Easy+Medium+Hard vs. Control (Table 1, Fig. 1), which represents the effect of the distance judgment task; and Medium vs. Easy (Table 2, Fig. 2), Hard vs. Medium (Table 3, Fig. 2) to examine the brain regions responsive to increment increases in difficulty for visuo-spatial judgments.

## Conflict of Interest

The authors (KMK, JRR, MAB, KMR) of this manuscript *(Functional magnetic resonance imaging data of incremental increases in visuo-spatial difficulty in an adult lifespan sample*) have no conflicts of interest to report.

## Figures and Tables

**Table 1:** Cluster peaks for the whole sample effect of distance judgment task [Easy, Medium, Hard vs. Control].

**A. Positive effect cluster-level**												
	**Cluster Label**		**BA**	***k***		***X***	***Y***		***Z***		***t*****-value**	***p***_**fwe**_
L/R superior occipital gyrus			18	7013		−6	−102		6		17.3	<.001
	and R precuneus		7			18	−66		45		14.46	
			7			27	−72		36		13.94	
L/R middle frontal gyrus			8	745		6	21		42		12.82	<.001
			6			−24	0		51		9.62	
R middle and inferior frontal			46	1409		45	36		18		12.54	<.001
	gyrus		13			33	21		−3		11.55	
			44			48	6		24		10.99	
L inferior frontal gyrus and			47	1305		−30	21		−3		11.6	<.001
	insula		6			−45	6		27		10.64	
			9			−45	27		27		8.38	
R middle frontal gyrus			6	323		30	6		54		10.05	<.001
R thalamus/caudate			50	88		18	−27		15		6.5	0.004
			48			18	−15		18		6.1	
			48			18	−3		18		5.76	
L thalamus/caudate			48	90		−18	−27		15		6.27	0.004
			48			−18	0		18		6.1	
			50			−15	−6		6		4.59	

**B. Negative effect cluster-level**												
		**Cluster Label**	**BA**	***k***		***X***		***Y***		***Z***	***t*****-value**	***p***_**fwe**_

							
R lingual gyrus			18	332		21		−90		−3	17.86	<.001
L inferior occipital gyrus			18	238		−21		−96		−9	16.21	<.001
R superior and middle			22	2342		60		−45		12	14.8	<.001
		temporal gyrus	39			60		−60		21	12.59	
			21			57		−9		−15	10.48	
L/R medial orbital and			10	3230		0		57		−3	12.61	<.001
		middle frontal gyrus;	8			−21		33		42	11.57	
		anterior cingulate	10			9		54		12	11.16	
L/R posterior and middle			23	1959		−3		−45		33	12.46	<.001
		cingulate gyrus	24			3		−21		39	10.6	
			23			−6		−27		39	10.5	
L middle occipital and			39	1429		−45		−75		39	11.8	<.001
		posterior parietal gyrus	39			−57		−63		27	10.44	
			19		−57			−69		9	10.4	
L middle temporal gyrus			21	278		−54		−9		−15	8.55	<.001
L orbital frontal gyrus			47	95		−33		33		−15	7.75	0.003
R orbital frontal gyrus			47	93		36		36		−12	7.06	0.003
			45			54		33		0	5.93	
R hippocampus			54	112		27		−12		−18	6.61	0.001
L cerebellum crus 2				39		−24		−81		−36	5.74	0.031
L hippocampus and fusiform			54	65		−27		−15		−18	5.61	0.008
		gyrus	37			−30		−33		−15	5.44	
L inferior temporal gyrus			20	41		−45		3		−36	5.1	0.022
			38			−33		3		−39	4.51	
R cerebellum crus 2				46		21		−87		−39	5.06	0.031
						30		−81		−36	4.48	

*Note. p*<.0001 uncorrected, cluster-level FWE *p*<.05 correction. BA=Brodmann׳s area.

**Table 2:** Cluster peaks for the whole sample effect of increasing difficulty from Easy to Medium.

**A. Increased activation from easy to medium cluster-level**								
	**Cluster Label**	**BA**	***k***	***X***	***Y***	***Z***	***t*****-value**	***p***_**fwe**_
L superior and middle		18	344	−9	−102	9	9.58	<.001
	occipital gyrus	18		−24	−93	15	7.68	
		18		−36	−78	3	4.66	
R cuneus and middle occipital		18	536	12	−96	15	9.51	<.001
	gyrus	18		27	−87	18	8.18	
		18		3	−81	−3	6.21	
L/R anterior cingulate gyrus		8	71	6	21	42	5.48	0.008
		32		−6	21	39	4.26	

**B. Deceased activation from easy to medium cluster-level**								
	**Cluster Label**	**BA**	***k***	***X***	***Y***	***Z***	***t*****-value**	***p***_**fwe**_

							
R inferior occipital gyrus		18	200	24	−93	−3	9.99	<.001
L inferior occipital gyrus and		18	379	−18	−93	−9	8.68	<.001
	cerebellum crus 1 & 2			−21	−78	−39	6.22	
				−33	−84	−30	6.15	
R middle temporal and gyrus		39	747	54	−60	21	7.63	<.001
	angular gyrus	37		66	−48	−3	5.59	
		39		48	−66	39	4.93	
L/R posterior cingulate gyrus		23	814	6	−45	30	6.32	<.001
	and precuneus	7		0	−57	45	6.31	
		7		−6	−57	66	6.25	
L middle and superior		8	215	−30	27	48	6.12	<.001
	frontal gyrus	8		−15	39	45	4.49	
		9		−36	36	36	4.11	
R cerebellum crus 1			358	54	−66	−33	6.02	<.001
				45	−72	−33	5.14	
				27	−81	−30	5.08	
L posterior parietal and		39	573	−39	−72	42	5.82	<.001
	middle temporal gyrus	39		−54	−45	30	5.57	
		39		−51	−63	18	5.13	
R middle frontal gyrus		8	74	27	30	45	5.35	0.007
R middle temporal gyrus		21	94	60	−9	−18	5.33	0.003
L inferior temporal gyrus		37	99	−57	−51	−6	5.2	0.003
L/R superior medial frontal		10	37	12	63	15	4.99	0.039

*Note. p*<.0001 uncorrected, cluster-level FWE *p*<.05. BA=Brodmann׳s area.

**Table 3:** Cluster peaks for the whole sample effect of increasing difficulty from Medium to Hard.

**A. Increased activation from medium to hard cluster-level**								
	**Cluster Label**	**BA**	***k***	***X***	***Y***	***Z***	***t*****-value**	***p***_**fwe**_
R inferior and superior		7	1315	42	−51	54	8.6	<.001
	parietal lobule	7		33	−60	54	8.43	
		40		42	−42	42	8.3	
R inferior frontal and		9	2220	48	33	21	8.38	<.001
	insula	44		48	9	24	8.32	
		13		30	24	−3	7.88	
L cerebellum crus 1 & 2			476	−9	−81	−33	8.29	<.001
				−33	−72	−48	7.77	
				−30	−66	−30	7.2	
R superior medial frontal gyrus		8	432	9	27	45	7.21	<.001
		8		3	33	39	6.98	
R lingual gyrus		18	37	18	−87	−6	6.75	0.04
L insular cortex		13	99	−33	21	−3	6.42	0.003
L inferior frontal gyrus		44	53	−57	21	30	5.45	0.019
L middle occipital gyrus		18	35	−36	−93	9	5.09	0.044
L orbitofrontal gyrus		47	41	−45	45	−6	5.06	0.033
L inferior and superior		40	141	−39	−48	48	4.81	0.001
	parietal lobule	39		−33	−57	48	4.79	
		7		−21	−66	51	4.6	

**B. Deceased activation from medium to hard cluster-level**								
	**Cluster Label**	**BA**	***k***	***X***	***Y***	***Z***	***t*****-value**	***p***_**fwe**_

							
L/R posterior and anterior		18	10811	0	−72	24	10.96	<.001
	medial wall and precuneus	10		−3	57	−3	9.75	
		23		9	−57	27	9.29	
L middle temporal and		39	1846	−42	−57	21	8	<.001
	angular gyrus	39		−42	−72	36	7.19	
		21		−54	−6	−18	6.93	
L superior frontal gyrus		8	222	−18	33	42	7.06	<.001
L orbital frontal gyrus		47	69	−27	36	−15	6.31	.014

*Note. p*<.0001 uncorrected, cluster-level FWE *p*<.05. BA=Brodmann׳s area.
